# A Rare Case of Male Breast Cancer Presenting as a Superficial Pigmented Nipple Tumor: The Importance of a Deep Excisional Biopsy and Immunohistochemical Analysis in Diagnosis

**DOI:** 10.7759/cureus.83353

**Published:** 2025-05-02

**Authors:** Ryo Nishiyori, Mayuko Goto-Umeki, Mizuho Fujinaga-Tada, Yohei Takumi, Atsushi Osoegawa, Riko Furukawa, Yuzo Oyama, Takashi Sakai, Yutaka Hatano

**Affiliations:** 1 Department of Dermatology, Oita University Faculty of Medicine, Yufu, JPN; 2 Department of Thoracic and Breast Surgery, Oita University Faculty of Medicine, Yufu, JPN; 3 Department of Diagnostic Pathology, Oita University Faculty of Medicine, Yufu, JPN

**Keywords:** dermoscopy, invasive ductal carcinoma, male breast cancer, nipple tumor, sweat gland carcinoma

## Abstract

Nipple lesions present diagnostic challenges due to overlapping clinical and histopathological features. A 65-year-old male presented with a black skin lesion on his right nipple, initially suspected to be malignant melanoma. Dermoscopy suggested basal cell carcinoma, while punch biopsy findings indicated sweat gland carcinoma with negative mammaglobin staining. However, total resection revealed cord-like invasion into the deep dermis near the mammary gland, and immunohistochemistry showed mammaglobin positivity, leading to a final diagnosis of solid-type invasive ductal carcinoma of the breast. This case highlights the diagnostic challenges of male nipple tumors and underscores the need for thorough histopathological and deep-layer assessment to distinguish breast cancer from cutaneous malignancies.

## Introduction

The diagnosis of nipple lesions encompasses a wide spectrum of conditions, often posing a diagnostic difficulty [1]. Invasive diagnostic procedures and repeated manipulations in this delicate area may be approached with caution due to potential functional and aesthetic consequences [2]. However, when malignancy is suspected, histopathological confirmation via biopsy is essential. Here, we present a rare case of male breast cancer (BC) manifesting as a superficial pigmented nipple tumor, which was difficult to differentiate from other cutaneous malignancies. Through this case, we discuss the differential diagnosis of nipple tumors.

## Case presentation

**Figure 1:**
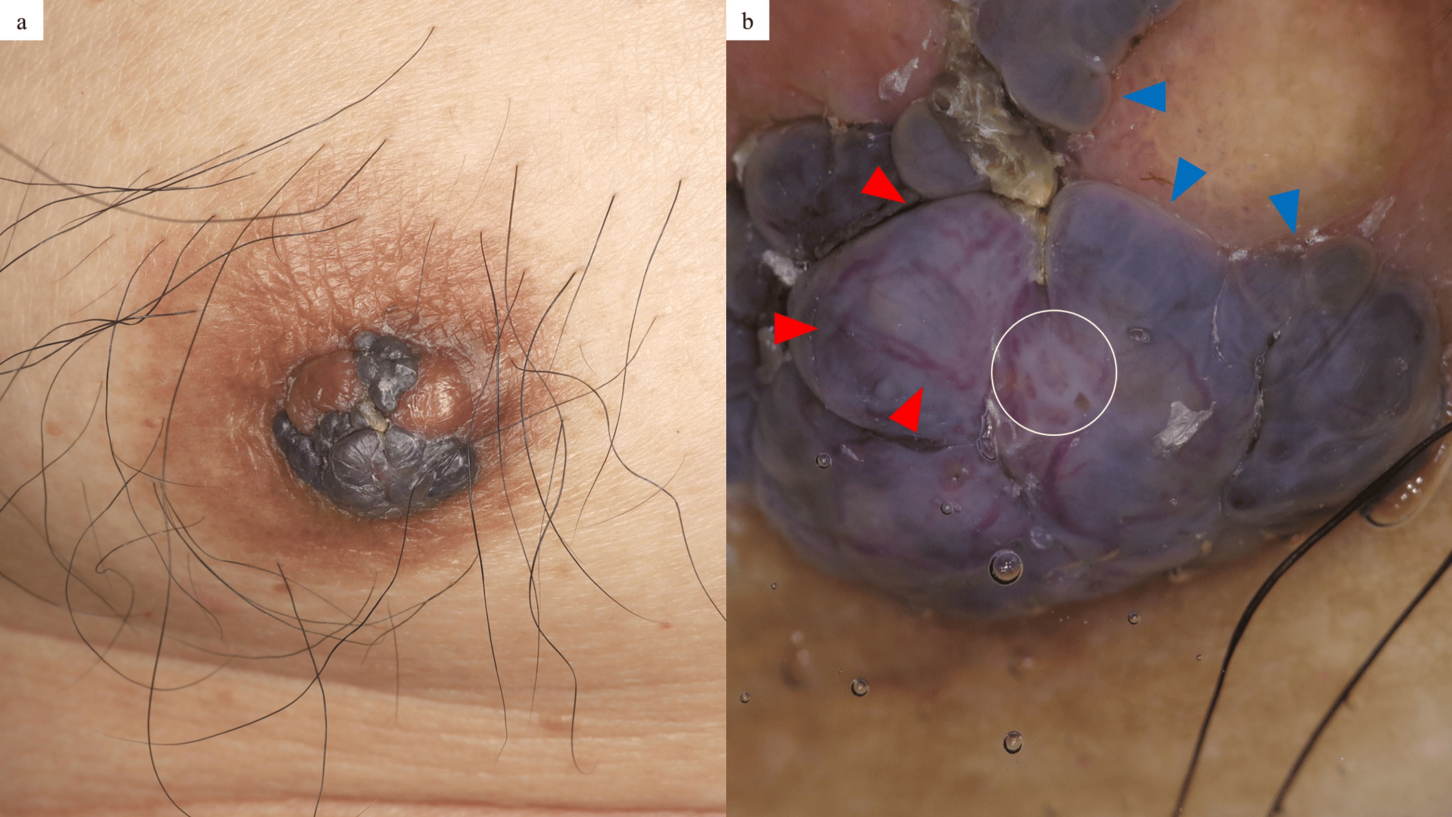
Cutaneous manifestation of the present case. A 12 × 10 mm multilobed black tumor was observed on the right nipple, with no palpable subcutaneous mass: (a). Dermoscopy showed blue-gray ovoid nests (blue arrowheads), shiny white structures (white circle), and arborizing vessels (red arrowheads) (b).

A 65-year-old male presented to a clinic specializing in breast diseases with a black skin lesion on his right nipple, first noticed a year earlier. The breast surgeon at the clinic suspected skin cancer, such as malignant melanoma (MM), and the patient was referred to our hospital’s Department of Dermatology for further evaluation. On examination, a 12 × 10 mm multilobed black tumor was observed on the right nipple, with no palpable subcutaneous mass (Figure 1a). Dermoscopy showed blue-gray ovoid nests, shiny white structures, and arborizing vessels (Figure 1b), features more suggestive of basal cell carcinoma (BCC) than MM [3].

**Figure 2:**
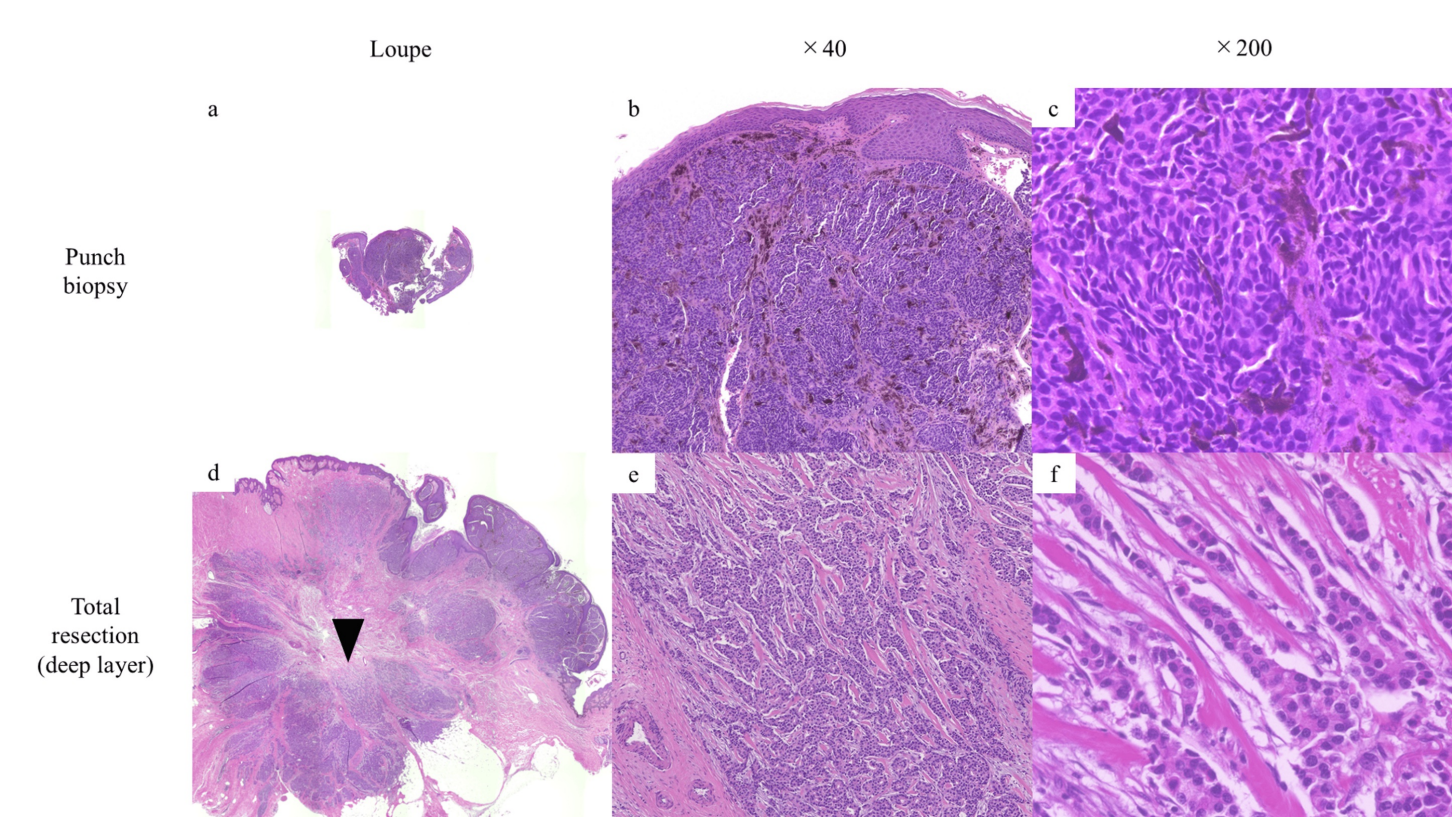
Histopathological findings of the punch biopsy and total resection. A punch biopsy revealed tumor cells forming nests in the superficial dermis with melanization: (a) loupe, (b) ×40, and (c) ×200. Total resection revealed tumor cells with pale eosinophilic cytoplasm and hyperchromatic round nuclei, displaying cord-like infiltration into the deep dermis near the mammary gland (black arrowhead): (d) loupe, (e) ×40, ad (f) ×200. (a-f: hematoxylin–eosin staining).

A punch biopsy revealed round tumor cells forming high-density nests in the superficial dermis with melanization without connection to the epidermis (Figures 2a-2c).

**Figure 3:**
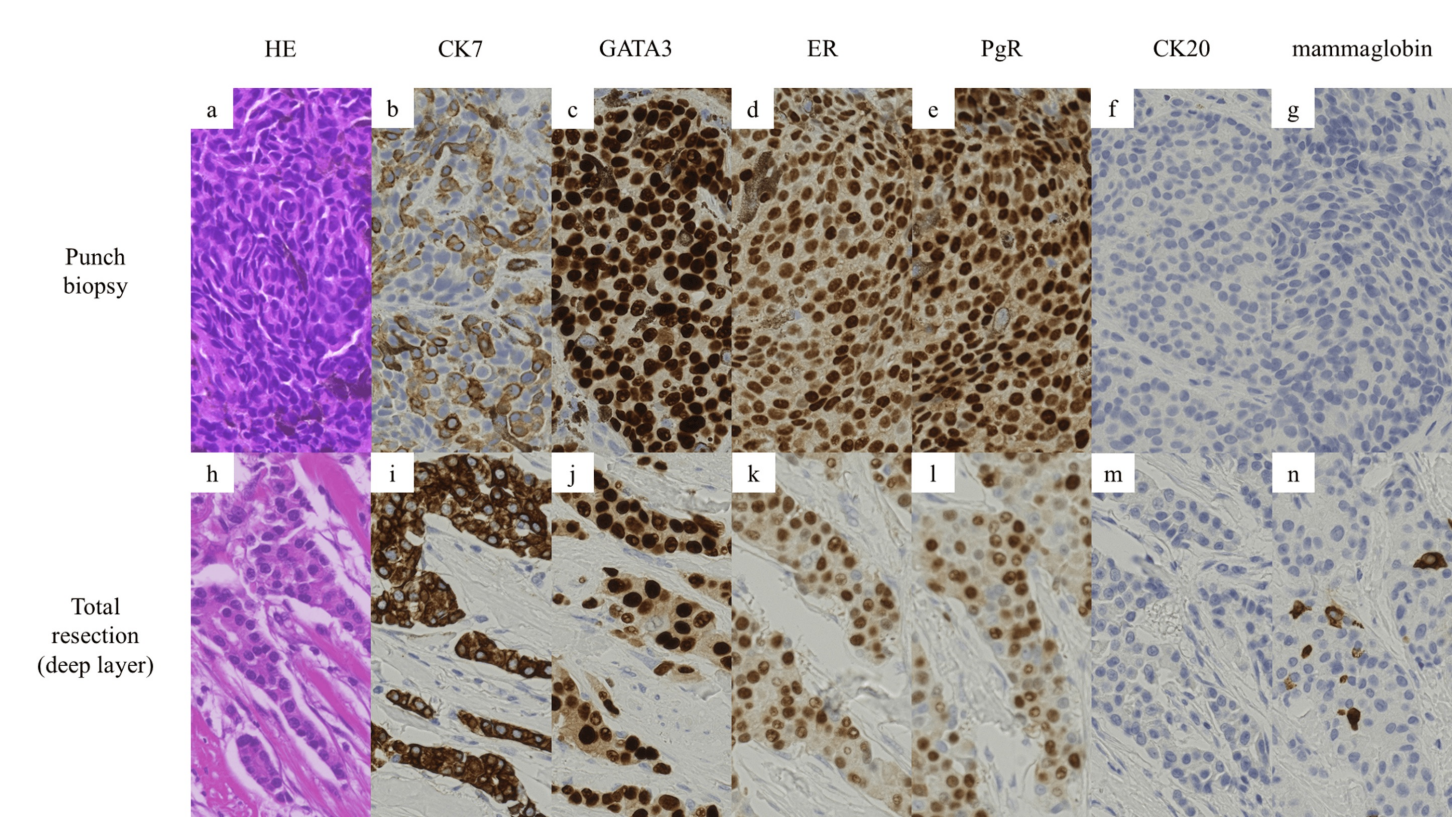
Immunohistochemical findings of the punch biopsy and total resection. A punch biopsy revealed the tumor cells were positive for cytokeratin 7 (CK7), GATA binding protein 3 (GATA3), estrogen receptor (ER), and progesterone receptor (PgR) and negative for cytokeratin 20 (CK20) and mammaglobin: (a-g) ×200. Total resection revealed that the tumor cells were positive for CK7, GATA3, ER, PgR, and mammaglobin, and negative for CK20: (h-n) ×200.

Immunohistochemically, the tumor cells were positive for cytokeratin 7 (CK7), GATA binding protein 3 (GATA3), estrogen receptor (ER), and progesterone receptor (PgR) and negative for cytokeratin 20 (CK20) and mammaglobin (Figures 3a-3g).

The tumor location and histopathological findings from hematoxylin-eosin staining suggested breast cancer (BC); however, the immunohistochemical profile - positive for CK7 and GATA3 and negative for mammaglobin - was more indicative of sweat gland carcinoma (SGC) than BC [4]. Despite these clinical and histological examinations, a definitive diagnosis could not be established; therefore, the tumor, including the nipple, was completely resected with a 2 mm safety margin. The histopathological examination of the total resection specimen revealed tumor cells with pale eosinophilic cytoplasm and hyperchromatic round nuclei, displaying cord-like infiltration into the deep dermis near the mammary gland, rather than being confined to the superficial layer (Figures 2d-2f). Immunohistochemistry showed that the tumor cells were positive for CK7, GATA3, ER, and PgR, and negative for CK20. Although only partially, the tumor cells exhibited positivity for mammaglobin (Figures 3h-3n). Notably, although mammaglobin was negative in the initial punch biopsy, which sampled a relatively superficial area, it was slightly positive in the deeply infiltrative lesion in the total resected specimen. Based on the tumor’s overall location, morphology, and immunostaining results for mammaglobin, we diagnosed the case as BC, specifically solid-type invasive ductal carcinoma. Subsequently, the patient was referred to the Department of Breast Surgery, where a right mastectomy and sentinel lymph node biopsy were performed. Although long-term follow-up data are not available at this point, the patient will undergo regular postoperative surveillance, including clinical exams and imaging, per standard protocols. The prognosis is favorable given the early stage (pT1cN0M0, stage I) and the initiation of adjuvant endocrine therapy [5].

## Discussion

The present case of male BC initially presented to a clinic specializing in breast diseases. However, the patient was referred to the Department of Dermatology and was ultimately diagnosed with BC through immunohistochemical analysis of the total resected specimen. This case highlights the diagnostic challenges associated with nipple tumors, particularly in males. Male BC is very rare, accounting for only 0.6% of all BC cases. Due to its rarity and lack of social recognition, male BC is often diagnosed at a more advanced stage than female BC [6]. Cutaneous involvement, such as nipple retraction or ulceration, is observed in 25% of male BC cases. However, reports of superficial tumor presentations are limited [7]. Additionally, primary pigmented BC is rare and can pose a diagnostic difficulty in clinical differentiation from MM [8]. Consequently, advanced male BC presenting as a superficial pigmented tumor rather than a subcutaneous mass can be particularly difficult to diagnose.

The dermoscopic findings were more suggestive of BCC than MM, specifically in terms of the presence of blue-gray ovoid nests, shiny white structures, and arborizing vessels. In general, the dermoscopic findings of blue-gray ovoid nests correspond to large, well-defined tumor nests with melanization in the dermis, while shiny white structures correspond to altered dermal collagen [3]. The histological findings of the present case showed large tumor nests in the superficial dermis with melanization, mimicking histologically BCC, potentially contributing to its BCC-like dermoscopic features of the present case. Moreover, although arborizing vessels are a key dermoscopic feature of BCC, they can also be observed in other conditions, such as nipple adenoma and apocrine mixed tumor [9]. Therefore, dermoscopic findings alone may not provide sufficient evidence to reliably distinguish BC from BCC. Dermoscopy primarily serves as a screening tool; thus, its findings should be interpreted with caution, with histopathological confirmation remaining essential for an accurate diagnosis. When a lesion is located anatomically near the mammary gland, clinicians may consider the possibility of BC, even if clinical morphology or dermoscopic findings are inconclusive.

A punch biopsy, which primarily sampled the superficial layer of the tumor, initially suggested SGC. SGC is also very rare, further complicating histological differentiation from male BC, which is similarly uncommon. SGC, particularly cutaneous apocrine carcinoma, typically forms tumor nests in the dermis and is often positive for CK7, GATA3, ER, and PgR and negative for CK20 and mammaglobin, which was consistent with this case [10]. However, subsequent total resection confirmed a cord-like invasion in the deep layer near the mammary gland on hematoxylin-eosin staining, with immunohistochemistry showing mammaglobin positivity, ultimately leading to the diagnosis of BC. Mentrikoski et al. reported that mammaglobin has a sensitivity of 45% and a specificity of 95% in differentiating metastatic breast carcinoma from SGC [4]. In a retrospective study of 234 male breast lesions, an ultrasound-guided core needle biopsy (CNB) demonstrated a sensitivity of 98.9% and a specificity of 100% for the detection of breast malignancy [11]. A punch biopsy, while minimally invasive and commonly used in dermatologic practice, may have limitations in evaluating nipple lesions, particularly when the pathology extends into deeper tissues near the mammary gland. A similar diagnostic challenge was reported by Moennich et al., in which a case of female BC was initially misdiagnosed as BCC following a shallow shave biopsy of a nipple lesion without any palpable mass or imaging abnormalities [12]. As demonstrated in the present case, superficial sampling may miss diagnostic features such as mammaglobin positivity or deep dermal invasion. While an ultrasound-guided CNB, which allows sampling of deeper tissues including the mammary gland, is more invasive, it may improve diagnostic accuracy in suspected cases of breast carcinoma and should be considered in selected cases. Although no standardized diagnostic algorithm exists specifically for ambiguous nipple lesions, a combined approach involving clinical evaluation, dermoscopy, imaging (including ultrasound), and histopathological assessment - preferably with deeper tissue sampling - should be considered when malignancy cannot be excluded.

## Conclusions

We reported a rare case of male BC that initially mimicked other cutaneous malignancies, including MM, BCC, and SGC, clinically and histopathologically. The final diagnosis of solid-type invasive ductal carcinoma was established only after complete excision and deep-layer histopathological assessment, including immunohistochemical staining for mammaglobin. This case highlights the diagnostic complexity of nipple lesions and underscores the importance of considering BC in the differential diagnosis, even in male patients.

Clinicians should be aware that superficial biopsy techniques may miss deeper malignancies, particularly in lesions located near the mammary gland. In such cases, a multidisciplinary diagnostic approach - including clinical examination, dermoscopy, imaging, and deep tissue biopsy - is essential for accurate diagnosis and appropriate treatment planning. Moreover, this case underscores the need for increased awareness and future research to establish standardized diagnostic protocols. Early recognition and accurate diagnosis of male BC remain critical for achieving favorable outcomes.
